# CD215+ Myeloid Cells Respond to Interleukin 15 Stimulation and Promote Tumor Progression

**DOI:** 10.3389/fimmu.2017.01713

**Published:** 2017-12-04

**Authors:** Shouheng Lin, Guohua Huang, Yiren Xiao, Wei Sun, Yuchuan Jiang, Qiuhua Deng, Muyun Peng, Xinru Wei, Wei Ye, Baiheng Li, Simiao Lin, Suna Wang, Qiting Wu, Qiubin Liang, Yangqiu Li, Xuchao Zhang, Yilong Wu, Pentao Liu, Duanqing Pei, Fenglei Yu, Zhesheng Wen, Yao Yao, Donghai Wu, Peng Li

**Affiliations:** ^1^Key Laboratory of Regenerative Biology, South China Institute for Stem Cell Biology and Regenerative Medicine, Guangzhou Institutes of Biomedicine and Health, Chinese Academy of Sciences, Guangzhou, China; ^2^Guangdong Provincial Key Laboratory of Stem Cell and Regenerative Medicine, South China Institute for Stem Cell Biology and Regenerative Medicine, Guangzhou Institutes of Biomedicine and Health, Chinese Academy of Sciences, Guangzhou, China; ^3^University of Chinese Academy of Sciences, Beijing, China; ^4^Department of Respiratory Medicine, Nanfang Hospital, Southern Medical University, Guangzhou, China; ^5^Department of Thoracic Oncology, Sun Yat-sen University Cancer Center, Guangzhou, China; ^6^Department of Thoracic Oncology, The Second Xiangya Hospital of Central South University, Changsha, China; ^7^Guangdong Zhaotai InVivo Biomedicine Co. Ltd., Guangzhou, China; ^8^Medical College, Institute of Hematology, Jinan University, Guangzhou, China; ^9^Guangdong Lung Cancer Institute, Medical Research Center, Guangdong General Hospital, Guangdong Academy of Medical Sciences, Guangzhou, China; ^10^Wellcome Trust Sanger Institute, Hinxton, United Kingdom; ^11^International Institute for Translational Chinese Medicine, Guangzhou University of Chinese Medicine, Guangzhou, China

**Keywords:** interleukin 15, CD215, IGF-1, patient-derived xenograft, lung cancer

## Abstract

Interleukin 15 (IL-15) regulates the development, survival, and functions of multiple innate and adaptive immune cells and plays a dual role in promoting both tumor cell growth and antitumor immunity. Here, we demonstrated that the *in vivo* injection of recombinant human IL-15 (200 µg/kg) or murine IL-15 (3 µg/kg) to tumor-bearing NOD-*SCID-IL2Rg−/−* (NSI) mice resulted in increased tumor progression and CD45+ CD11b+ Gr-1+ CD215+ cell expansion in the tumors and spleen. In B16F10-bearing C57BL/6 mice model, we found that murine IL-15 has antitumoral effect since the activation and expansion of CD8+ T cells with murine IL-15 treatment. But no enhanced or reduced tumor growth was observed in mice when human IL-15 was used. However, both murine and human IL-15 promote CD45+ CD11b+ Gr-1+ CD215+ cells expansion. In xenograft tumor models, CD215+ myeloid cells, but not CD215*−* cells, responded to human IL-15 stimulation and promoted tumor growth. Furthermore, we found that human IL-15 mediated insulin-like growth factor-1 production in CD215+ myeloid cells and blocking IGF-1 reduced the tumor-promoting effect of IL-15. Finally, we observed that higher IGF-1 expression is an indicator of poor prognosis among lung adenocarcinoma patients. These findings provide evidence that IL-15 may promote tumor cell progression *via* CD215+ myeloid cells, and IGF-1 may be an important candidate that IL-15 facilitates tumor growth.

## Introduction

Immune-stimulatory cytokines can be exploited to treat human cancer (1, 2). Many prior studies have suggested the importance of interleukin 15 (IL-15) as a regulator for immune cells (3–6). IL-15 exhibits broad activity and induces the differentiation and proliferation of lymphocytes (1, 7) and myelocytes (8, 9). The studies of mutant mice deficient in IL-15 or IL-15Rα suggest that IL-15 play a multifaceted role in the development and control of the immune system (10–13). Thus, IL-15 has become a promising candidate for tumor immunotherapy; IL-15 administration is used to bolster immune responses (14, 15) and augment the tumor immune surveillance (16, 17). However, there are other reports indicating that IL-15 protects tumor cells from apoptosis (18, 19). Studies which have used immunodeficient mice show that IL-15 can be pro-tumorigenic through the promotion of tumor growth, invasion, and metastasis (20), overexpression of IL-15 promotes the development of large granular lymphocytic leukemia (21, 22). However, whether IL-15 is required for the development or suppression of inflammation-induced cancer is poorly understood.

Interleukin 15 signals are delivered *via* a heterotrimeric receptor complex (23). Along with its specific IL-15Rα subunit (CD215), which is required for high-affinity IL-15 binding, the IL-15R complex also contains a β subunit (IL-15/IL-2Rβ or CD122), which IL-15 shares with IL-2, and a common γ chain (γc or CD132). IL-15 signaling in natural killer (NK) cells and CD8+ T cells occurs *via* a *trans* presentation, where accessory cells, such as macrophages or dendritic cells (DCs), present IL-15-bound IL-15Rα in *trans* to NK cells or CD8+ T cells expressing IL-15/IL-2Rβ and γc. Specifically, IL-15 can signal *via* CD215/JNK to drive RANTES production by myeloid cells (24). IL-15 has been reported to induce myeloid cells to produce cytokines and chemokines, such as IL-2, TNFα, and IFNα (25–31).

Tumor infiltration by a variety of immune cells, including cytotoxic T cells, regulatory T cells, NK cells, monocytes, DCs, and macrophages, is a common feature of many cancers (32, 33). Although tumor infiltration by cytotoxic lymphocytes is generally correlated with a favorable outcome (34), substantial evidence has shown that myeloid cells, such as monocytes, DCs, and macrophages, can instead promote tumorigenesis by supplying cytokines (such as CCL2, IGF-1, and EGF) that stimulate tumor proliferation, tissue invasion, and/or angiogenesis (35, 36). The role of these cells in promoting tumor progression was primarily discovered *via* studies of spontaneous and transplanted murine tumor models with normal immune systems (33). Great advances in the understanding of the roles played by myeloid cells in tumor progression have depended on the observation of their systematic progression in immunodeficient host mice, such as immunodeficient non-obese diabetic (NOD)-SCID mice and NOD/LtSz-SCID IL-2rγ−/− (NSG or NOG) mice (37, 38). However, it remains to be investigated whether and how IL-15 might enhance cancer-promoting inflammation.

Myeloid cells have been reported to mediate cell growth and survival through IGF-1 (39, 40). Other reports have also indicated that the IGF-1 signaling pathway may be implicated in several cancers (41, 42). However, whether the tumor-associated myeloid cells participate in tumor progression through IGF-1 is still elusive. Furthermore, the function of IL-15 in this biological process remains unknown.

Here, we investigated whether and how IL-15 contributes to myeloid cell-mediated tumor progression. Our findings demonstrate that IL-15 induced CD215+ myeloid cell proliferation and that these myeloid cells promoted tumor growth. Furthermore, IGF-1 expression was elevated in CD215+ myeloid cells and influenced tumor progression; additionally, its expression level was correlated with poor patient survival. Thus, our results suggest that CD215+ myeloid cells respond to IL-15 and promote cancer progression, and IGF-1 may be an important candidate that IL-15 facilitates tumor growth.

## Materials and Methods

### Mice

Animal experiments were performed in the Laboratory Animal Center of the Guangzhou Institutes of Biomedicine and Health (GIBH), and all animal procedures were approved by the Animal Welfare Committee of GIBH. NOD-*SCID-IL2Rg−/−* (NSI) mice were derived at the GIBH (43). C57BL/6 mice were purchased from Vital River Laboratory Animal Technology Co. (Beijing). All mice were maintained in specific-pathogen-free cages and provided autoclaved food and water. Protocols were approved by the relevant Institutional Animal Care and Use Committee.

### Cell Lines

Two human non-small cell lung carcinoma cell lines (A549 and H1299, both adenocarcinomas) and a human prostate cancer cell line (DU145) were cultured in RPMI-1640 (Gibco, New York, NY, USA) supplemented with 10% fetal bovine serum (FBS; Biochrom, Australia) and passaged at 80% confluence.

A549 cells expressing GFP and luciferase were cultured in RPMI-1640 (Gibco, New York, NY, USA), supplemented with 10% FBS (Biochrom, Australia) and passaged at 80% confluence.

Murine melanoma cells (B16F10) were cultured in Dulbecco’s Modified Eagle Medium (DMEM, Gibco, New York, NY, USA) supplemented with 10% FBS and passaged at 80% confluence. All cells were cultured at 37°C in 5% CO_2_ and a normal level of O_2_.

The A549, H1299, and DU145 cells used in this study were obtained from the ATCC between 2014 and 2015 and kept at low passage numbers for experimental use. New A549, H1299, and DU145 cells were purchased from the ATCC after they were cultured for 6 months. *Mycoplasma* infection was detected with a Mycoplasma Detection Kit (Lonza, Rockland, ME, USA). We routinely screened for *Mycoplasma* and discarded the *Mycoplasma*-positive cells.

### Cytokines and Antibody

For flow cytometric analysis, antibodies were purchased from eBioscience, including mouse CD45-APC, mouse CD215-PE, mouse CD122-PE, and mouse CD132-PE, mouse CD45-Percp-Cy5.5, mouse CD11b-FITC, and mouse Gr-1-APC.

For culture and animal studies, recombinant human IL-15 used in the *in vivo* studies was a kind gift from Professor Donghai Wu from the GIBH in China; recombinant human IL-15 (Peprotech, Rocky Hill, CT, USA) was used in coculture experiments. Recombinant murine IL-15 (Peprotech, Rocky Hill, CT, USA) was used in the *in vivo* studies and *in vitro* culture experiment. Anti-human IL-15 (ct2nu, eBioscience, USA), anti-mouse IL-15Rα antibody (sc-9172, IL-15Rα antibody, Santa Cruz Biotechnology, USA), and anti-mouse IGF-1 (ab9572, Abcam, Cambridge, UK) were used in coculture experiments and in animal studies. Antibody against human IGF-1 receptor (ab39675, Abcam, Cambridge, UK) was used in Immunohistochemical staining.

### Animal Studies

Cancer cell line-derived subcutaneous mouse model A549 human lung cancer cells (5 × 10^5^), H1299 human lung cancer cells (5 × 10^5^), or DU145 human prostate cancer cells (5 × 10^5^) were subcutaneously injected into the hind flank of 6- to 8-week-old NSI mice. The mice were split into groups of at least six mice and treated with either control (PBS) or 200 µg/kg human recombinant IL-15 once per week by intravenous injection until the mice were sacrificed. Tumor volumes were calculated after caliper measurements of two axes using the formula: (width·width·length)/2 where width < length. The mice were sacrificed 4–8 weeks after tumor cell injection.

B16F10 murine melanoma cells (1 × 10^6^) were subcutaneously injected into the hind flank of 6- to 8-week-old C57BL/6 mice. Mice were treated with either control (PBS) or 200 µg/kg human recombinant IL-15 once per week by intravenous injection until the mice were sacrificed. Tumor volumes were calculated after caliper measurements of two axes using the formula: (width·width·length)/2 where width < length. The mice were sacrificed 5 weeks after tumor cell injection.

For murine IL-15 test, A549 human lung cancer cells (5 × 10^5^) were subcutaneously injected into the hind flank of 6- to 8-week-old NSI mice; B16F10 murine melanoma cells (1 × 10^6^) were subcutaneously injected into the hind flank of 6- to 8-week-old C57BL/6 mice. The mice were split into groups of at least six mice and treated with either control (PBS) or 3 µg/kg recombinant murine IL-15 once per week by intravenous injection until the mice were sacrificed. Tumor volumes were calculated after caliper measurements of two axes using the formula: (width·width·length)/2 where width < length. The mice were sacrificed 4 weeks after tumor cell injection.

A549 human lung cancer cells (5 × 10^5^) were mixed with CD45+ CD11b+ Gr-1+ CD215+ cells (5 × 10^4^) or CD45+ CD11b+ Gr-1+ CD215*−* cells (5 × 10^4^) and subcutaneously injected into NSI mice. Mice were split into groups of at least six mice and treated with either control (PBS) or 200 µg/kg human recombinant IL-15 or 200 µg/kg human recombinant IL-15 plus 1 mg/kg anti-IL-15 antibody (ct2nu, eBioscience, USA) once per week by intravenous injection until the mice were sacrificed. Tumor volumes were calculated after caliper measurements of two axes using the formula: (width·width·length)/2 where width < length. The mice were sacrificed 4 weeks after tumor cell injection. The recombinant human IL-15 used in the *in vivo* studies was a kind gift from Professor Donghai Wu from the GIBH in China.

Patient-derived xenograft (PDX) mouse model—all human lung cancer tissues were obtained from Sun Yat-sen University Cancer Center (SYSUCC) in Guangzhou, China. Written informed consent was obtained from each patient. The tissue used in this study was approved by the committee for the ethical review of research involving human subjects at SYSUCC. The tissue was collected fresh and was immediately dissected, minced into tissue blocks at approximately 3 mm in diameter, and placed in Matrigel matrix (Corning, NY, USA) with antibiotics. Then, the human cancer tissue blocks were subcutaneously transplanted into the NSI mice within 30 min after resection. The mice were observed daily for tumor growth. The tumors were removed and passaged once the tumors reached 1.5 cm^3^ in diameter. On the third passage and once tumors reached 100 mm^3^ (as measured by calipers), treatments with (PBS) or 200 µg/kg human recombinant IL-15 once per week by intravenous injection were initiated. Tumor volumes were calculated after caliper measurements of two axes using the formula: (width·width·length)/2 where width < length. The mice were sacrificed 6 weeks after tumor cell injection.

For the IGF-1 animal study, A549 human lung cancer cells (5 × 10^5^) were mixed with CD45+ CD11b+ Gr-1+ CD215+ cells (5 × 10^4^) or CD45+ CD11b+ Gr-1+ CD215*−* cells (5 × 10^4^) and subcutaneously injected into NSI mice. Mice were split into groups of at least six mice and treated with either control (PBS) or 200 µg/kg human recombinant IL-15 or 200 µg/kg human recombinant IL-15 plus 500 µg/kg IGF-1 neutralizing antibody (ab9572, Abcam, Cambridge, UK) once per week by intravenous injection. Tumor volumes were calculated after caliper measurements of two axes using the formula: (width·width·length)/2 where width < length. The mice were sacrificed 4 weeks after tumor cell injection.

### Cell Culture Experiments

For the IL-15 culture experiment, 1,000 tumor cells were cultured in 96-well plates with RMPI-1640 supplemented with 10% FBS with or without 500 ng/mL human IL-15. Tumor cell numbers were measured by Cell Counting Kit-8 (CCK-8, DOJINDO, Japan) at the indicated times.

For the coculture experiment, a total of 30 A549-GFP-luciferase cells were precultured in a 96-well plate with RPMI-1640 supplemented with 10% FBS overnight. Before coculturing A549-GFP-luciferase cells with the indicated mouse cells, A549-GFP-luciferase cells from each well of the 96-well plate were washed with PBS and counted under a microscope. The wells containing between 58 and 62 cells were chosen for use in the coculture experiment. Cells were cultured with RPMI-1640 supplemented with 10% FBS plus 20 ng/mL GM-CSF. The IL-15 culture conditions were as follows: cells were cultured in RPMI-1640 supplemented with 10% FBS plus 20 ng/mL GM-CSF and 500 ng/mL human IL-15 (Peprotech, Rocky Hill, CT, USA) with or without 5 µg/mL anti-human IL-15 (ct2nu, eBioscience, USA) and with or without 3 µg/mL anti-mouse IL-15Rα antibody (sc-9172, IL-15Rα antibody, Santa Cruz Biotechnology, USA); and the CD215+ or CD215*−* cells (2 × 10^4^) were purified by flow cytometric sorting from A549 tumor-bearing mice 45 days after tumor cell injection. Anti-IGF-1 culture conditions were as follows: cells were cultured in RPMI-1640 supplemented with 10% FBS plus 20 ng/mL GM-CSF and 500 ng/mL human IL-15 (Peprotech, Rocky Hill, CT, USA) with or without 5 µg/mL anti-human IL-15 (ct2nu, eBioscience, USA) and with or without 3 µg/mL anti-mouse IGF-1 (ab9572, Abcam, Cambridge, UK); and the CD215+ or CD215*−* cells (2 × 10^4^) were purified by flow cytometric sorting from A549 tumor-bearing mice. Tumor cell numbers were measured by a luciferase assay at the indicated time. The recombinant human IL-15 and mouse GM-CSF used for the *in vitro* culture experiments were purchased from Peprotech (Peprotech, Rocky Hill, CT, USA).

For IGF-1 culture experiments, 1,000 A549 or H1299 cells were cultured in a 96-well plate with RMPI-1640 supplemented with 10% FBS with or without 10 ng/mL murine IGF-1 (Peprotech, Rocky Hill, CT, USA) or 3 µg/mL anti-mouse IGF-1 (ab9572, Abcam, Cambridge, UK). Tumor cell numbers were measured by Cell Counting Kit-8 (CCK-8, DOJINDO, Japan) at the indicated times according to the manufacturer’s instructions.

### Luciferase Assay

Luciferase activity in A549-GFP-luciferase cells was assessed using the Promega Luciferase Assay System according to the manufacturer’s instructions (Promega). Washed cells were digested with 40 µL of 0.25% trypsin at 37°C for 5 min. d-Luciferin substrate was diluted with RPMI-1640 containing 10% FBS at a concentration of 150 µg/mL. The digested cells were resuspended with 100 µL of d-Luciferin-medium mix. A 100-µL volume of cell suspension was transferred to a 96-well white plate. Photon emission was measured with a Veritas™ Microplate Luminometer (Promega).

### ELISA

Mouse IGF-1 ELISA kits were purchased from R&D Systems (Minneapolis, MN, USA). All ELISAs were performed using cell-free supernatants according to the manufacturer’s instructions. Briefly, the concentration of the measured cytokine was calculated as the average of triplicate samples (each adjusted for background signal and normalized to blank wells) and subsequently converted to a total final concentration upon comparison to the standards provided in the kit. All samples were analyzed in duplicate at dilutions of fourfold to eightfold that adhered to the dynamic range of the assay.

### Flow Cytometry

Solid tissues were mechanically chopped with scalpels and placed in culture medium (DMEM with 5% FBS, 0.5 mg/mL collagenase A, 0.2 mg/mL hyaluronidase V, and 0.02 mg/mL DNase I). They were digested for 45 min at 37°C. The resulting suspensions were resuspended in PBS, and the cells were pelleted at 300 r.c.f. for 3 min.

All antibodies were purchased from eBioscience unless otherwise noted. Flow cytometric analysis was performed using Accuri C6 or LSRFORTESSA (BD Biosciences, San Jose, CA, USA). Mouse CD45-APC, mouse CD215-PE, mouse CD122-PE, and mouse CD132-PE were used for both the analysis and live cell sorting. 4′, 6-diamidino-2-phenylindole (DAPI; 0.1 µg/mL final concentration; Invitrogen) was used to distinguished between live and dead cells.

For FACS separation, splenocytes and tumor cells from NSI mice and C57BL/6 mice were labeled with mouse CD45-Percp-Cy5.5, mouse CD215-PE, mouse CD11b-FITC, and mouse Gr-1-APC antibodies for 20 min at 4°C and were sorted with a MoFlo Astrios cell sorter (Beckman Coulter, Indianapolis, IN, USA). Post-sort analysis usually indicated purities of >95%.

### Gene Expression Analysis

The sequencing reads were mapped to the mouse RefSeq-RNA reference sequence (downloaded from http://hgdownload.cse.ucsc.edu/downloads) using the FANSe 2 algorithm (available from http://bioinformatics.jnu.edu.cn/software/fanse2/) with the parameters *−*L85 *−*E3 *−*U0 *−*S1015. Alternative splice variants were merged. Genes with at least 10 mapped reads were considered to be reliably detected genes. These genes were further quantified using count values, which were raw counts of sequencing reads. The count values were imported into the DESeq software package to calculate the up/downregulation of genes among untreated CD215*−* cells, untreated CD215+ cells, CD215*−* cells treated with IL-15, and CD215+ cells treated with IL-15. For the RNA-Seq analysis, all count values of mouse genes with more than a twofold change were imported into the DAVID database (available from http://david.abcc.ncifcrf.gov/home.jsp). The up- or downregulated genes were identified by filtering the RNA-Seq data with the following cutoff criteria: a twofold change in expression level and *p* < 0.05. Gene enrichment terms from the Gene Ontology (GO) analysis were obtained using DAVID.^1^ Pathway analysis was performed using GeneMANIA.^2^

### Quantitative Real-time PCR Analysis

Total RNA was extracted using TRIzol reagent (Invitrogen, Burlington, ON, USA). The concentration of the total RNA was measured using a Nanodrop 2000 (Thermo Scientific, MA, USA). One microgram of total RNA was reverse transcribed using TransScript II First-Strand cDNA Synthesis SuperMix (Transgen, Beijing). Quantitative real-time PCR was performed in triplicate using SYBR Green PCR Master Mix (Roche Bioscience) in a 20-µL reaction with predesigned mouse-specific primers. The primer sequences are as follows.

**Table:** 

Gene	GenBank accession ID	Primers	Amplicon size (bp)
*Bcl-2*	NM_009741	5′-ATGCCTTTGTGGAACTATATGGC-3′(forward)	120
		5′-GGTATGCACCCAGAGTGATGC-3′(reverse)	

*Bcl-xl*	NM_001289716	5′-GACAAGGAGATGCAGGTATTGG-3′(forward)	124
		5′-TCCCGTAGAGATCCACAAAAGT-3′(reverse)	

*Sox4*	NM_009238	5′-CGGCTGCATCGTTCTCTCC-3′(forward)	166
		5′-CGCTTCACTTTCTTGTCGGC-3′(reverse)

*Lef1*	NM_010703	5′-AACGAGTCCGAAATCATCCCA-3′(forward)	165
		5′-GCCAGAGTAACTGGAGTAGGA-3′(reverse)	

*Igf-1*	NM_010512	5′-AGACAGGCATTGTGGATGAG-3′(forward)	136
		5′-TGAGTCTTGGGCATGTCAGT-3′(reverse)	

*Pdgfa*	NM_008808	5′-TGGCTCGAAGTCAGATCCACA-3′(forward)	133
		5′-TTCTCGGGCACATGGTTAATG-3′(reverse)	

*Bdnf*	NM_007540	5′-TCATACTTCGGTTGCATGAAGG-3′(forward)	137
		5′-AGACCTCTCGAACCTGCCC-3′(reverse)	

*Fgf13*	NM_010200	5′-CTCATCCGGCAAAAGAGACAA-3′(forward)	140
		5′-TTGGAGCCAAAGAGTTTGACC-3′(reverse)	

### Immunohistochemical Staining and Analyses

Paraffin-embedded sections were deparaffinized, stained with an antibody against IGF-1 receptor (ab39675, Abcam, Cambridge, UK), and imaged with a microscope (DMI6000B; Leica Microsystems, Wetzlar, Germany).

### Data Mining

The associations between IGF-1 mRNA expression and clinical features and outcomes of lung cancer were obtained by Oncomine Cancer Microarray database analysis.^3^ Data were retrieved from the Oncomine website and reanalyzed in GraphPad software to show the correlation between of IGF-1 and patient survival. Additional details of the study are available at Oncomine.

### Statistical Analysis

Statistical analyses used are detailed in the figure legends. One-way ANOVA or unpaired two-tailed Student’s *t*-test was used to establish the statistical significance using GraphPad Prism (GraphPad Software). For survival analyses, Kaplan–Meier plots were drawn and statistical differences evaluated using the log-rank (Mantel–Cox) test. Statistical significance set at *p* < 0.05.

## Results

### IL-15 Promotes Tumor Growth in Immunodeficient Mice

**Figure 1:**
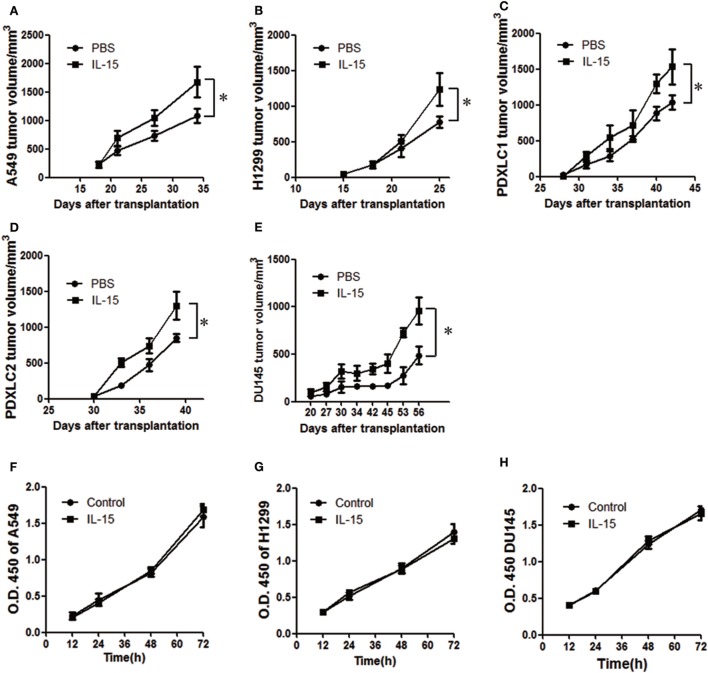
Interleukin 15 (IL-15) promotes tumor growth *in vivo*. Tumor growth curve showing enhanced A549 **(A)**, H1299 **(B)**, PDXLC1 **(C)**, PDXLC2 **(D)**, and DU145 **(E)** tumor growth in NSI mice following the injection of IL-15 (*n* = 6). Tumor growth curve showing the lack of enhanced A549 **(F)**, H1299 **(G)**, and DU145 **(H)** tumor growth *in vitro* with IL-15 treatment. Data are mean ± SD of one representative experiment. Similar results were observed in three independent experiments. *p < 0.05, **p < 0.01, and ***p < 0.001; ns, not significant. Unpaired two-tailed *t*-test. Statistics on tumor growth curve **(A–E)**, *p*-values are significance for measurements of tumor volume before sacrificing mice.

Numerous prior studies have suggested the importance of IL-15 in cancer therapy (1, 7). However, there are other reports indicating that IL-15 is associated with cell proliferation in several cancers (20). To explore the possibility that IL-15 promotes lung cancer cell growth, we established cell line-derived xenograft and PDX models with NOD-*SCID-IL2Rg−/−* (NSI) mice. IL-15 mentioned in this study is human IL-15 unless otherwise specified. We first tested the IL-15 dosage *in vivo* in tumor-bearing mice. Considering the short half-life of exogenous IL-15 in mice, we used the high dose of IL-15 as previous studies described (16, 17), and we found enhanced tumor growth in the 5 µg IL-15 (200 µg/kg) group (Figure S1A in Supplementary Material). All mice survived the tested IL-15 dosages, suggesting that the IL-15 dosage we used was no toxic to the mice. IL-15 treatment significantly increased tumor cell growth in the A549, H1299 cell line-derived xenograft model, and two lung cancer PDX model in NSI mice (Figures 1A–D, Figures S1B–E in Supplementary Material; Table 1). We also found that IL-15 treatment significantly increased DU145 prostate tumor cell growth *in vivo* (Figure 1E; Figure S1F in Supplementary Material). However, increased tumor growth was not observed in mice with intact immune systems (Figures S1G,H in Supplementary Material). To further explore the protumoral effect of IL-15 in a lower dose as it used in clinical trial (44), we treated B16F10-bearing C57BL/6 mice and A549-bearing NSI mice with 3 µg/kg murine IL-15. Murine IL-15 showed the antitumoral activity in mice with intact immune systems (Figures 1I,J in Supplementary Material); however, we still observed an increased tumor growth in immunodeficient mice with lower dose of murine IL-15 (Figures S1K,L in Supplementary Material). We further tested whether IL-15 could directly promote the proliferation of tumor cells. To our surprise, culturing cells with IL-15 did not result in increased tumor cell growth (Figures 1F–H). These results suggest that cells in the NSI mice likely respond to IL-15, which promotes tumor growth. Since the development of T cells, B cells, and NK cells is impaired in NSI mice (43), we hypothesized that a subpopulation of myeloid cells in NSI mice may respond to IL-15 stimulation and promote cancer progression.

**Table 1:** Information of patients whose samples were used to establish patient-derived xenograft models.

Sample	Gender	Age	Histology
PDXLC1	Female	47	Lung adenocarcinoma
PDXLC2	Male	62	Lung adenocarcinoma

### IL-15 Promotes CD215+ Myeloid Cell Proliferation

Previous studies have suggested that IL-15 binds to three main receptors: IL-15 receptor α (IL-15Rα, CD215), IL-2 receptor β (IL-2Rβ, CD122), and IL-2 receptor γc (IL-2Rγc, CD132). In NSI mice, the γc receptor is functionally deficient. Because IL-15 binds to its specific α receptor chain with high affinity, we investigated whether CD215+ cells respond to IL-15 in NSI mice. Compared to B6 mice with intact immune systems, NSI mice exhibit impaired development of T cells, B cells, and NK cells. Thus, we performed flow cytometric analysis to confirm that almost all CD215+ cells in the tumor and spleen of NSI tumor-bearing mice are CD45+ CD11b+ Gr-1+ myeloid cells (Figure S2A,B Supplementary Material).

**Figure 2:**
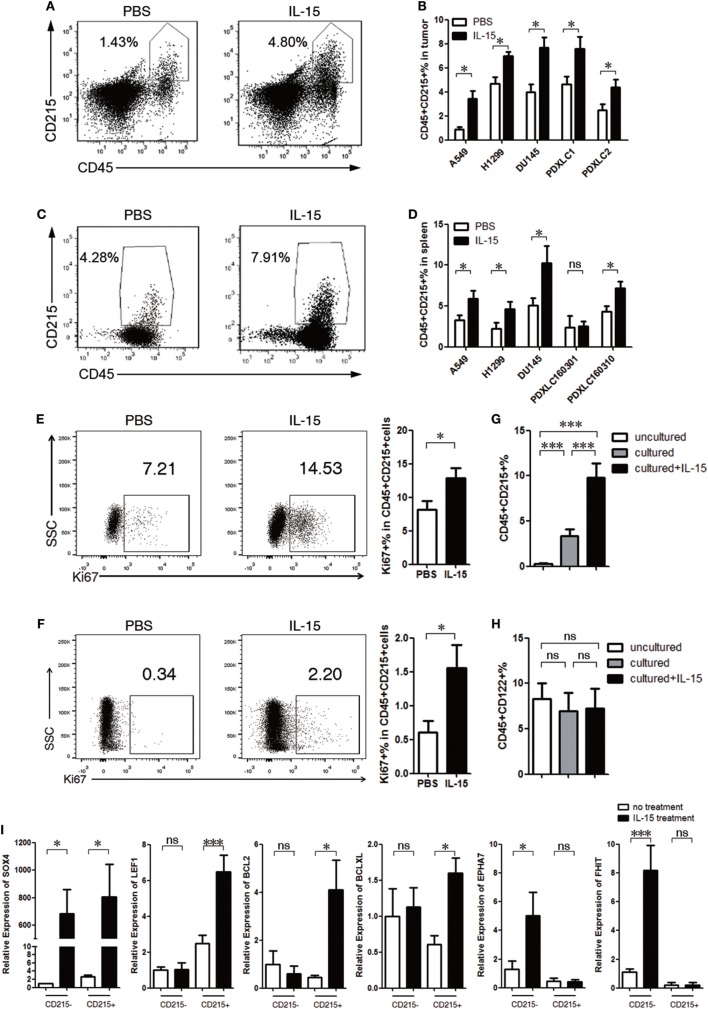
Interleukin 15 (IL-15) promotes CD215+ myeloid cell expansion. **(A–D)** CD215+ cells expanded in tumor-bearing mice after IL-15 treatment. Mice were injected with IL-15 once per week and sacrificed 4–8 weeks after tumor cell injection. **(A)** Representative FACS plots showing the percentages of tumor CD45+ CD215+ leukocytes in PDXLC2 tumor-bearing mice. Left: PBS; right: IL-15. **(B)** Summary of the FACS analysis of mouse tumor CD215+ cell frequencies among CD45+ leukocytes from A549, H1299, DU145, PDXLC1, and PDXLC2 tumor-bearing mice *(n* = 6 each). **(C)** Representative FACS plots showing the percentages of splenic CD45+ CD215+ leukocyte cell frequencies in PDXLC2 tumor-bearing mice. Left: PBS; and right: IL-15. **(D)** Summary of the FACS analysis of splenic CD215+ cell frequencies among CD45+ leukocytes from A549, H1299, DU145, PDXLC1, and PDXLC2 tumor-bearing mice (*n* = 6 each). Representative FACS plots and summary showing the percentages of Ki67+ cells among CD45+ CD215+ cells from the spleen **(E)** and tumors **(F)** of A549 tumor-bearing mice. Splenocytes were plated on a 24-well plate at a density of 1 × 10^6^ cells/mL with or without human recombinant IL-15. After 24 h, CD45+ CD215+ cells **(G)** and CD45+ CD122+ cells **(H)** frequencies among leukocytes were measured by FACS (*n* = 6 each). **(I)** qRT-PCR analysis of the indicated mRNA in CD215+ myeloid or CD215*−* cells treated with or without IL-15. The results were normalized to the glyceraldehyde 3-phosphate dehydrogenase (GAPDH) mRNA levels and are presented as the mean ± SEM (*n* = 3). Data are mean ± SD of one representative experiment **(B,D–H)**. Similar results were observed in three independent experiments. Data are mean ± SEM (*n* = 3) **(I)**. *p < 0.05, **p < 0.01, and ***p < 0.001; ns, not significant. One-way ANOVA **(G–I)**, and unpaired two-tailed *t*-test **(B,D–F)**.

To examine the effect of IL-15 on the proliferation of CD215+ myeloid cells, we performed flow cytometric analysis of both mouse tumor and splenic cells from tumor-bearing mice 4–8 weeks after tumor implantation. We found that the percentage of CD215+ myeloid cells was significantly increased after IL-15 treatment (Figures 2A–D). The percentage of Ki67+ CD45+ CD215+ cells was also increased after IL-15 treatment (Figures 2E,F). In contrast, the percentage of CD45+ CD215*−* cells was unchanged after IL-15 treatment (Figures S2C,D Supplementary Material). We also found that the proportion of CD45+ CD122+ cells in tumor-bearing mice was unchanged after IL-15 treatment (Figures S2E,F Supplementary Material). These observations were further confirmed by culturing splenic cells from tumor-bearing mice *in vitro*; the percentage of CD45+ CD215+ cells were significantly increased after IL-15 stimulation, whereas the percentage of CD45+ CD122+ cells remained unchanged (Figures 2G,H).

To further investigate whether IL-15 could induce the proliferation of CD215+ myeloid cells in mice with intact immune systems, B16F10 tumor cells were implanted into C57BL/6 mice, which were treated with or without IL-15. The expansion of CD45+ CD215+ cells was not increased with IL-15 treatment (Figure S2G in Supplementary Material). However, the myeloid subset of CD45+ CD11b+ Gr-1+ CD215+ cells was expanded following IL-15 treatment (Figure S2H in Supplementary Material), supporting the hypothesis that IL-15 promotes CD215+ myeloid cell proliferation.

We also examined the effect of lower dose of murine IL-15 on the proliferation of CD215+ myeloid cells. We performed flow cytometric analysis of peripheral blood, splenic cells, and tumor cells from B16F10-bearing C57BL/6 mice and A549-bearing NSI mice with 3 µg/kg murine IL-15 4 weeks after tumor implantation. We could not observe tumor growth advantage in B16F10-bearing C57BL/6 mice with murine IL-15 treatment; it likely due to the expansion and activation of CD8+ T cells upon murine IL-15 treatment (Figures S3A–D in Supplementary Material). However, the CD45+ CD11b+ Gr-1+ CD215+ cells were expanded following murine IL-15 treatment (Figure S3E in Supplementary Material). In addition, lower dose of murine IL-15 also expanded the myeloid CD215+ population in A549-bearing NSI mice (Figure S3F in Supplementary Material). Furthermore, we *in vitro* cultured splenocytes from A549-bearing NSI mice, the percentage of CD45+ CD215+ cells were significantly increased after 10 ng/mL murine IL-15 stimulation (Figure S3G in Supplementary Material). Therefore, IL-15 is able to promote CD215+ myeloid cell proliferation in the dose in clinical use.

To further investigate how IL-15 promotes CD215+ myeloid cell proliferation, we performed real-time PCR and found that the expression of genes related to anti-apoptosis (*Bcl-2* and *Bcl-xl*) and cell proliferation (*Lef1* and *Sox4*) was upregulated (Figure 2I), suggesting that IL-15 exerts effects on the proliferation and survival of CD215+ myeloid cells. Despite high expression of *Sox4* in CD215*−* cells after IL-15 treatment, we could not observe the growth advantage of CD215*−* cells. However, we found that CD215*−* cells had high expression of *Epha7* and *Fhit* after IL-15 treatment. Previous studies indicated that Epha7 negatively regulates cell proliferation and trigger for apoptosis in a caspase-3-dependent manner (45) and Fhit is tumor-suppressor gene in relation to induction of apoptosis and cell cycle alteration (46). Therefore, IL-15 may have a dual effect on CD215*−* cells.

### CD215+ Myeloid Cells Respond to IL-15 and Promote Tumor Growth

**Figure 3:**
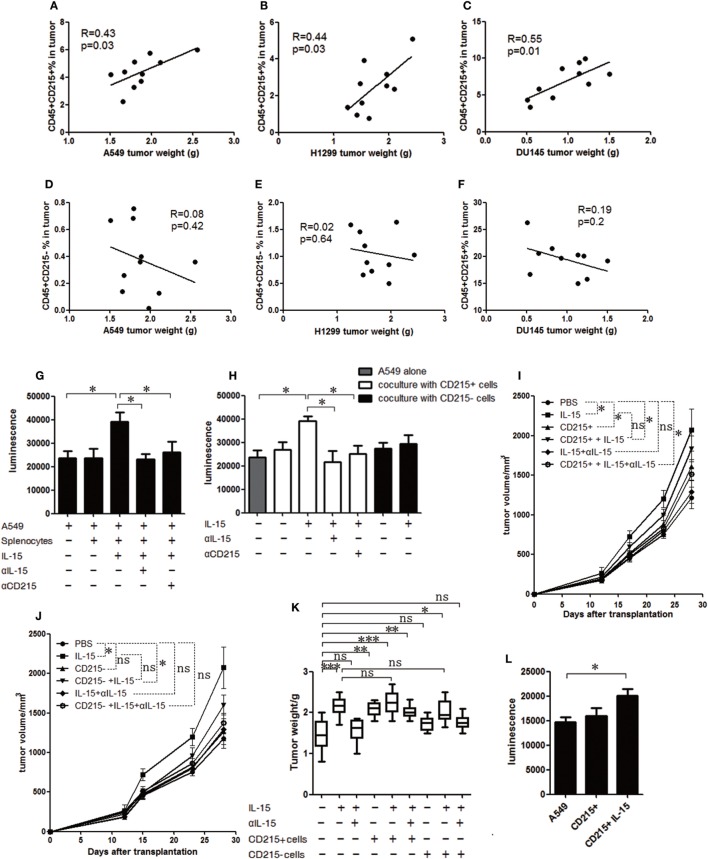
CD215+ myeloid cells respond to interleukin 15 (IL-15) and promote tumor growth. Analysis of the correlations between tumor volume and tumor-infiltrated CD45+ CD215+ cells in A549 **(A)**, H1299 **(B)**, and DU145 **(C)** tumor-bearing mice (*n* = 10). Analysis of the correlations between tumor volume and tumor-infiltrated CD45+ CD215*−* cells in A549 **(D)**, H1299 **(E)**, and DU145 **(F)** tumor-bearing mice (*n* = 10). **(G)** Quantification of A549-GFP-luciferase cell growth *in vitro* by luciferin after culture in basal medium or coculture with splenocytes; splenocytes plus IL-15; splenocytes plus anti-IL-15 antibody (αIL-15) and IL-15; or splenocytes plus anti-CD215 antibody (αCD215) and IL-15 for 120 h. **(H)** Quantification of A549-GFP-luciferase cell growth *in vitro* by luciferin after culture in basal medium or coculture with CD215+ cells; CD215+ cells plus IL-15; CD215+ cells plus anti-IL-15 antibody (αIL-15) and IL-15; CD215+ cells plus anti-CD215 antibody (αCD215) and IL-15; CD215*−* cells; or CD215*−* cells plus IL-15 for 120 h. Splenocytes were collected from A549 tumor-bearing mice 45 days after tumor cell injection. CD45+ CD11b+ Gr-1+ CD215+ cells and CD45+ CD11b+ Gr-1+ CD215*−* cells were purified by flow cytometric sorting. **(I)** Tumor growth curve showing enhanced A549 tumor growth in NSI mice with the addition of CD215+ cells; CD215+ cells plus IL-15; and CD215+ cells plus IL-15 and anti-IL-15 antibody (αIL-15) to the tumor cells (*n* = 8). **(J)** Tumor growth curve showing the lack of enhanced A549 tumor growth in NSI mice with the addition of CD215*−* cells or CD215*−* cells plus IL-15 and anti-IL-15 antibody (αIL-15) to the tumor cells (*n* = 8). **(K)** A549 tumor weight in NSI mice that were treated as indicated. CD215+ cells or CD215*−* cells were co-transplanted with A549 at day 0. IL-15 was intravenously injected once per week until the mice were sacrificed (*n* = 8). **(L)** Quantification of A549-GFP-luciferase cell growth *in vitro* by luciferin after culture in basal medium or coculture with CD45+ CD11b+ Gr-1+ CD215+ (CD215+) splenocytes or CD45+ CD11b+ Gr-1+ CD215+ splenocytes plus IL-15 (CD215+ IL-15) for 120 h. The splenocytes were isolated from tumor-bearing C57BL/6 mice. Data are mean ± SD of one representative experiment **(G–L)**. Similar results were observed in three independent experiments (*p < 0.05, **p < 0.01, and ***p < 0.001; ns, not significant). One-way ANOVA. Statistics on tumor growth curve **(I,J)**, *p*-values are significance for measurements of tumor volume before sacrificing mice.

To identify whether CD215+ myeloid cells can mediate tumor progression, we examined CD215+ myeloid cells and CD215*−* cells in the tumor microenvironment. We found that the percentage of CD215+ myeloid cells, but not CD215*−* cells, in the tumor were positively correlated with the tumor volume (Figures 3A–F). Then, to address whether CD215+ myeloid cells can directly promote tumor growth, we cocultured A549-GFP-luciferase cells and splenic cells from tumor-bearing mice and assessed the tumor cell expansion rates. The cultured tumor cells grew faster after stimulating the splenic cells with IL-15, whereas no growth advantage was observed when IL-15 or CD215 was blocked with specific neutralizing antibodies (Figure 3G). Next, we sorted CD45+ CD11b+ Gr-1+ CD215+ and CD45+ CD11b+ Gr-1+ CD215*−* cells from the spleen of tumor-bearing mice and cultured them with A549-GFP-luciferase cells. We found that CD215+ myeloid cells, but not CD215*−* myeloid cells, endowed a growth advantage to A549-GFP-luciferase cells following stimulation with IL-15 (Figure 3H). Furthermore, to assess whether CD215+ myeloid cells contribute to tumor progression *in vivo*, we purified CD45+ CD11b+ Gr-1+ CD215+ and CD45+ CD11b+ Gr-1+ CD215*−* cells from tumor-bearing mice, mixed them with A549 tumor cells, and injected the mixtures into NSI mice. Mice were treated with IL-15 or anti-IL-15 antibody once per week by intravenous injection until the mice were sacrificed. We found that CD215+ myeloid cells, but not CD215*−* myeloid cells, significantly increased tumor growth (Figures 3I–K).

Next, to extend our findings in immunodeficient mice to mice with intact immune systems, we sorted CD45+ CD11b+ Gr-1+ CD215+ myeloid cells from C57BL/6 mice bearing B16F10 tumors and performed a coculture experiment. We found the CD215+ myeloid cells from tumor-bearing wild-type mice also promoted tumor cell growth (Figure 3L). Therefore, CD215+ myeloid cells respond to IL-15 and promote tumor growth.

### IGF-1 May Be a Potential Candidate That IL-15 Facilitates Tumor Growth

**Figure 4:**
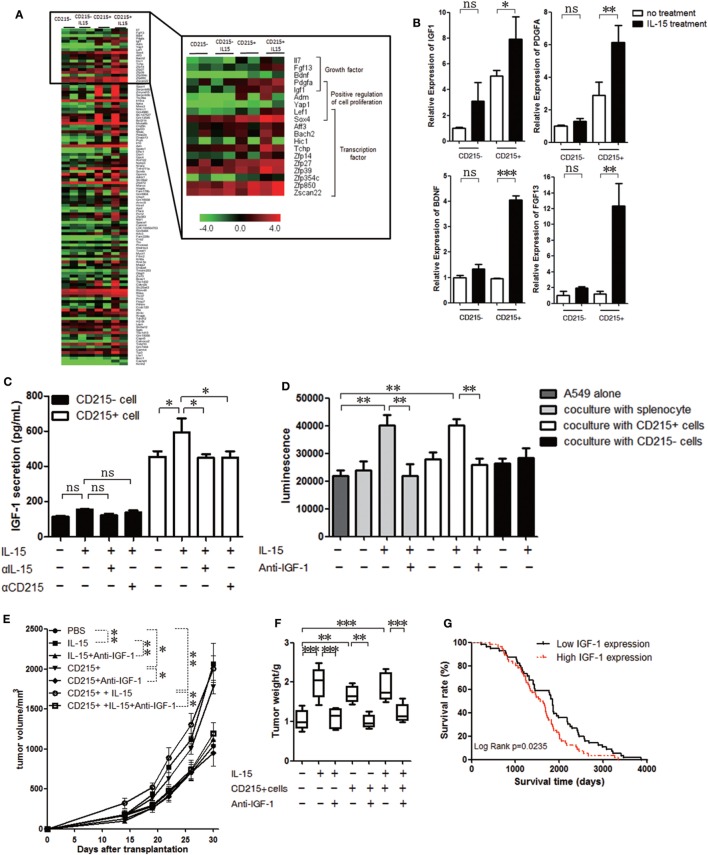
IGF-1 promotes tumor cell proliferation. **(A)** Unsupervised hierarchical cluster analysis of gene expression levels in CD215+ myeloid or CD215*−* cells treated with or without interleukin 15 (IL-15). Red: increased expression; green: decreased expression. **(B)** qRT-PCR analyses of gene expression in CD215+ myeloid or CD215*−* cells treated with or without IL-15. The results were normalized to the GAPDH mRNA levels and are presented as the mean + SEM (*n* = 3). **(C)** ELISA of the culture medium supernatant from CD215+ myeloid or CD215*−* cells 72 h after treatment with IL-15, anti-IL-15 antibody (αIL-15), or anti-CD215 antibody (αCD215). **(D)** Quantification of A549-GFP-luciferase cell growth *in vitro* by luciferin after culture in basal medium or coculture with splenocytes; splenocytes plus IL-15; splenocytes plus anti-IGF-1 antibody and IL-15; CD215+ cells; CD215+ cells plus IL-15; CD215+ cells plus anti-IGF-1 antibody and IL-15; CD215*−* cells; or CD215*−* cells plus IL-15 for 120 h. Splenocytes were collected from A549 tumor-bearing mice 45 days after tumor cell injection. CD45+ CD11b+ Gr-1+ CD215+ cells and CD45+ CD11b+ Gr-1+ CD215*−* cells were purified by flow cytometric sorting. **(E)** Tumor growth curve showing enhanced A549 tumor growth in NSI mice with the addition of IL-15; CD215+ cells; and CD215+ cells plus IL-15 to the tumor cells, but lack of enhanced A549 tumor growth with the addition of anti-IGF-1 antibody (*n* = 6). **(F)** A549 tumor weight in NSI mice that were treated as indicated. CD215+ cells were co-transplanted with A549 at day 0. IL-15 and anti-IGF-1 antibody were intravenously injected once per week until the mice were sacrificed (*n* = 6). **(G)** Kaplan–Meier survival curves of human lung adenocarcinoma patients with high (*n* = 59) versus low IGF-1 (*n* = 167) expression. Data are mean ± SEM (*n* = 3) **(B,C)**. Data are mean ± SD of one representative experiment **(D,E)**. Similar results were observed in three independent experiments (*p < 0.05, **p < 0.01, and ***p < 0.001; ns, not significant). One-way ANOVA **(B–E)** and log-rank Mantel–Cox test **(G)**. Statistics on tumor growth curve **(E)**, *p*-values are significance for measurements of tumor volume before sacrificing mice.

To assess potential alterations in CD215+ myeloid cells after IL-15 treatment, we FACS-purified CD45+ CD11b+ Gr-1+ CD215+ and CD45+ CD11b+ Gr-1+ CD215*−* cells from the tumor tissue of tumor-bearing mice and performed transcriptome profiling of these populations from two independent mice with or without IL-15 treatment using RNA-Seq. Gene expression analysis revealed a large number of upregulated genes after IL-15 treatment (Figure 4A; Table S1 in Supplementary Material). We further performed GO analyses on the upregulated genes and observed that the enriched term was related to the functions of cell growth (Figure S4A in Supplementary Material). We found that compared with untreated CD215+ myeloid cells, a set of growth factor genes was highly expressed in IL-15-treated CD215+ myeloid cells (Figure 4A). High expression of the growth factors was also confirmed by real-time PCR (Figure 4B). Considering the important role of IGF-1 in tumor progression, we examined whether IGF-1 was responsible for the tumor growth advantage with IL-15 stimulation. IGF-1 receptor expression was confirmed in all tumor cells used in the analysis by RT-PCR and immunohistochemistry (Figures S4B,C in Supplementary Material). To examine whether IGF-1 production was related to IL-15 stimulation, CD45+ CD11b+ Gr-1+ CD215+ and CD45+ CD11b+ Gr-1+ CD215*−* cells were sorted from tumor tissue and cultured for 72 h in medium with IL-15, anti-IL-15 antibody (αIL-15) or anti-CD215 antibody (αCD215). CD215+ cells cultured in medium containing IL-15 secreted high amounts of IGF-1, but IGF-1 production was decreased when IL-15 or CD215 was blocked (Figure 4C). We also obtained similar result when we cultured CD215+ or CD215*−* cells with murine IL-15 (Figure S4D in Supplementary Material). These results indicate that CD215+ myeloid cells produce IGF-1 following IL-15 stimulation.

Several studies showed that anti-IGF-1 inhibits tumor growth (47–49). To further investigate whether IGF-1 facilitate tumor growth in our study, we sorted CD45+ CD11b+ Gr-1+ CD215+ and CD45+ CD11b+ Gr-1+ CD215*−* cells from the spleen of tumor-bearing mice and cultured them with A549-GFP-luciferase cells with IL-15 or IL-15 plus anti-IGF-1 antibody. The tumor cell growth advantage was not observed when IGF-1 was blocked with antibody (Figure 4D; Figure S4E in Supplementary Material). We further assessed whether CD215+ myeloid cells contribute to tumor progression *via* IGF-1 *in vivo*. We purified CD45+ CD11b+ Gr-1+ CD215+ from tumor-bearing mice, mixed them with A549 tumor cells, and injected the mixtures into NSI mice. The protumoral effect of CD215+ myeloid cells was not observed when we treated mice with anti-IGF-1 antibody (Figures 4E,F). Therefore, we conclude that IGF-1 may be an important candidate that IL-15 facilitates tumor growth.

Finally, we analyzed human lung adenocarcinoma gene expression in lung cancer patients (50) to determine whether IGF-1 expression can predict patient outcomes. We found that higher IGF-1 expression levels were correlated with significantly shorter survival time (Figure 4G). Therefore, IGF-1 could be a negative prognostic factor in lung adenocarcinoma patients. Additionally, these results indicate that myeloid CD45+ CD215+ cells play an important role in lung cancer progression under IL-15 stimulation.

## Discussion

Although a critical role has been shown for IL-15 in regulating the development, survival, and activation of NK, T, and B cells (51), IL-15 also has effects on other components of the immune system, including neutrophils (52, 53), mast cells (9, 54), and DCs (55, 56). Adoptive immunotherapy using chimeric antigen receptors targeting tumor-associated antigens is recognized as an effective therapy for tumors (57–59). The critical role of IL-15, in regulating the most fundamental functions of lymphocytes, makes it an exciting candidate for immune therapy (18). In addition, IL-15 is reported to associate with the effectiveness of immune therapy for patients with relapsed lymphoma (60). However, there are other reports indicating that IL-15 also participates in the growth and survival of malignant cells in hematopoietic malignancies and solid tumors (20, 21, 61, 62). The protumoral activity of IL-15 may be underestimated since most of the analyses to study IL-15 function are using immune-sufficient mice and focus on its effect on CD8 T cells or NK cells (12, 13, 16, 17). However, it has been reported that GM-CSF and IL-15 fusokine is an immunosuppressor and allow engraftment of allogeneic B16F10 in immunocompetent mice (62). Other studies also show the effect of IL-15 on cancer initiation and progression in immunodeficient mice (20–22). In this study, we use immunodeficient mice lacking T cells, B cells, and NK cells to study IL-15 and identify an important role for IL-15 in promoting lung cancer growth. Our study further revealed that CD215+ myeloid cells, but not CD215*−* cells, respond to IL-15 and promote tumor growth. Therefore, the results from our current study suggest that CD215+ myeloid cells play an important role in the tumor microenvironment and in cancer progression.

Interleukin 15 has been reported to prevent apoptosis and promote the activation, proliferation, and survival of target cells that express the IL-15-receptor complex (10). It has also been reported that IL-15 triggers the antiapoptotic pathway and upregulates Bcl-2 or Bcl-xl expression to prevent cell apoptosis (63–65). Although most IL-15 signaling studies focus on its *trans* presentation, IL-15 also utilizes a distinct signaling pathway in mast cells (66). Previous studies have demonstrated that SOX4 and LEF1 play important roles in cell survival and proliferation (67–69). Our findings are consistent with many of these results and demonstrate that IL-15 promotes CD215+ myeloid cell growth. In addition, IL-15 mediates CD215+ myeloid cell growth through the expression of Bcl-2, Bcl-xl, Sox4, and Lef1. Pathway analysis of the upregulated genes in IL-15-stimulated CD215+ cells indicated that the Wnt signaling pathway may be critical for CD215+ cell proliferation (Figure 4F in Supplementary Material). Additional studies are required to determine whether the previously reported IL-15 signaling pathways (24, 64, 70) contribute to CD215+ myeloid cell growth.

The CD215+ myeloid cells we describe are CD45+ CD11b+ Gr-1+ cells that can promote tumor growth. The results from our studies suggest that this subset of myeloid cells also exists in mice with intact immune systems. Both human IL-15 (200 µg/kg) and murine IL-15 (3 g/kg) treatment promote CD215+ myeloid cells in B16F10-bearing C57BL/6 mice, and these CD215+ myeloid cells were sorted and shown to promote tumor growth in cell culture systems. In addition, CD11b and Gr-1 expression is usually used to identify myeloid-derived suppressor cells (MDSCs) in tumor-bearing mice (71). Additional studies are required to investigate whether these CD215+ myeloid cells are subpopulations of MDSCs and to determine their potential effects on the immune system.

Xenograft model was used to study the IGF-1 function in cancer development (72). Other reports have also indicated that IGF-1 plays an important role in tumor progression and may be a predictor of cancer risk (73–76). Our findings demonstrate that IL-15 enhances IGF-1 production in CD215+ myeloid cells; blocking IGF-1 significantly reduced the protumoral effect of IL-15. Since IGF-1 is also produced by other types of cells, such as macrophage and epithelial cells (77, 78), our results cannot exclude the possibility that IGF-1 plays a role independent of CD215+ cells. However, our results shed light on the possibility that IGF-1 may be an important candidate that IL-15 facilitates tumor growth. In addition to IGF-1, PDGFA (79, 80), BDNF (81, 82), and FGF13 (83, 84) have also been shown to contribute to tumor progression in previous studies. Thus, these growth factors may also participate in the tumor-promoting effect of CD215+ myeloid cells. However, the mechanism underlying this biological process is indeed interesting and requires further investigation.

It has been reported that murine cells respond to human IL-15, but most of the studies focus on the effect of IL-15 on CD8 T cells or NK cells (14–16, 85). Our study provides the evidence that murine CD215+ myeloid cells also respond to human IL-15. Because of the short half-life and reduced activity of exogenous IL-15 in mice, we used the high dose of human IL-15 (200 µg/kg) as previous studies described (16, 17). The highest dose administered in human clinical trials is 3 µg/kg (44), we also treated tumor-bearing mice with 3 µg/kg murine IL-15. We observed the enhanced tumor growth in immunodeficient mice with human IL-15 treatment. Both human and murine IL-15 promoted CD215+ myeloid cell expansion in immunocompetent or immunodeficient mice. In B16F10-bearing C57BL/6 mice model, we found that murine IL-15 has antitumoral effect since the activation and expansion of CD8+ T cells with murine IL-15 treatment. But we could not observe enhanced or reduced tumor growth in mice when human IL-15 was used. It is probably because of the short half-life and reduced activity of human IL-15 in mice, the immune systems are not fully activated. Therefore, whether IL-15 promotes human CD215+ cell expansion and whether IL-15 facilitates tumor growth in clinical application require further investigation.

In summary, our study indicates that IL-15 elicits a distinct tumor-promoting effect from tumor-associated myeloid cells. Our findings may also support the targeting of CD215+ myeloid cells in cancer therapy.

## Ethics Statement

This study was carried out in accordance with the recommendations of the Laboratory Animal Center of the GIBH. The protocol was approved by the Animal Welfare Committee of GIBH.

## Author Contributions

YX, SL, and GH contributed to the conception and design; the collection and/or assembly of data; data analysis and interpretation; and manuscript writing. WS, YJ, QD, MP, XW, QL, and WY contributed to the provision of study material or patients and the collection and/or assembly of data. BL, SL, SW, and QW provided animal care and administrative support. YL, XZ, YW, PtL, DP, FY, ZW, and DW contributed to the conception and design of the study. YY and PL contributed to the conception and design of the study; data analysis and interpretation; manuscript writing; and the final approval of the manuscript and provided financial support. All authors read and approved the final manuscript.

## Conflict of Interest Statement

The authors declare that the research was conducted in the absence of any commercial or financial relationships that could be construed as a potential conflict of interest.

## Footnotes

^1^https://david.ncifcrf.gov/.

^2^http://www.genemania.org.

^3^http://www.oncomine.org.
