# Supplementary figures and images for: CD215+ Myeloid Cells Respond to Interleukin 15 Stimulation and Promote Tumor Progression
Source: Front Immunol. 2017 Dec 4;8:1713. doi: 10.3389/fimmu.2017.01713 (PMC5722806; doi:10.3389/fimmu.2017.01713)

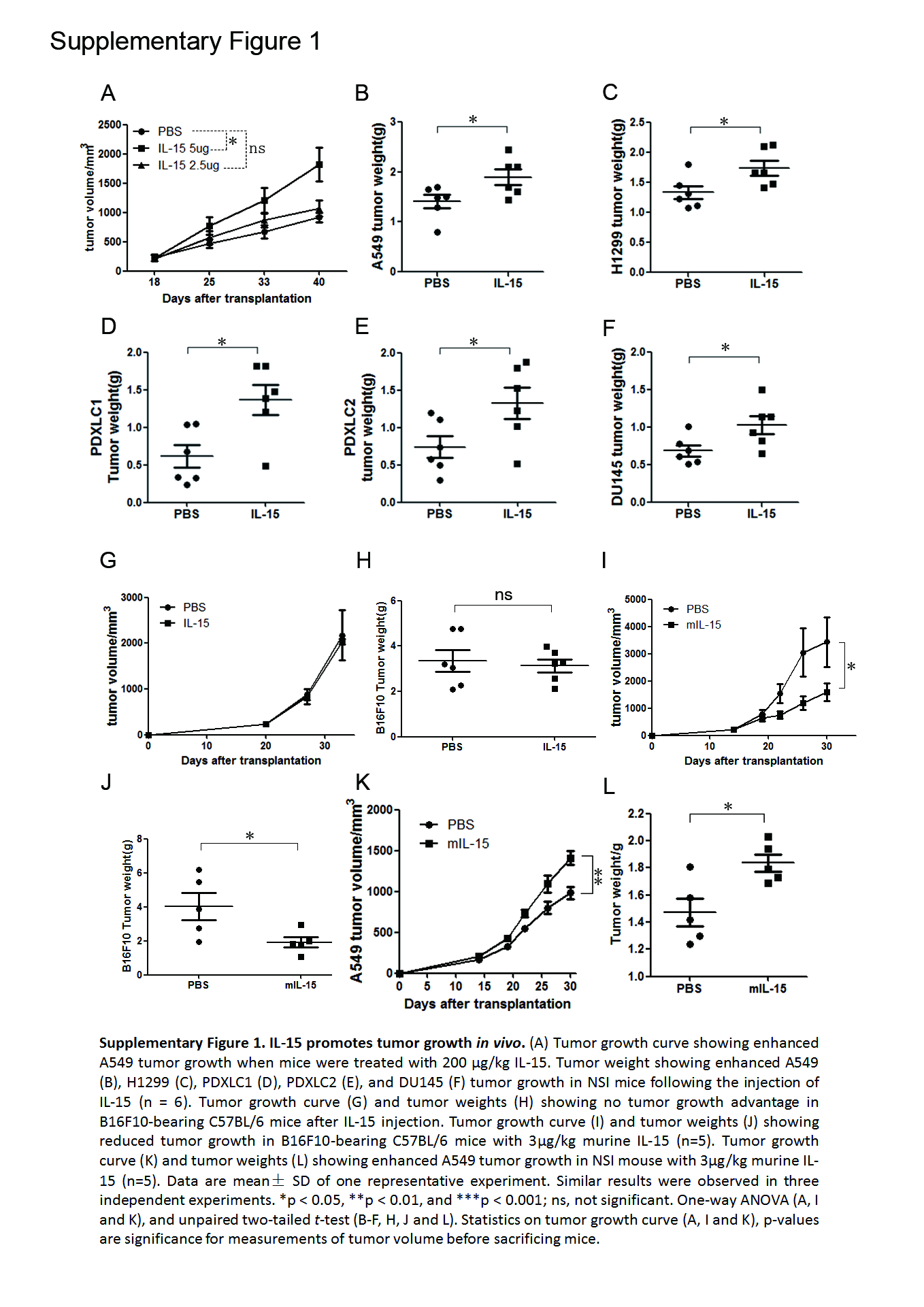

Supplement: Supplementary file 1 [file image_1.tif]

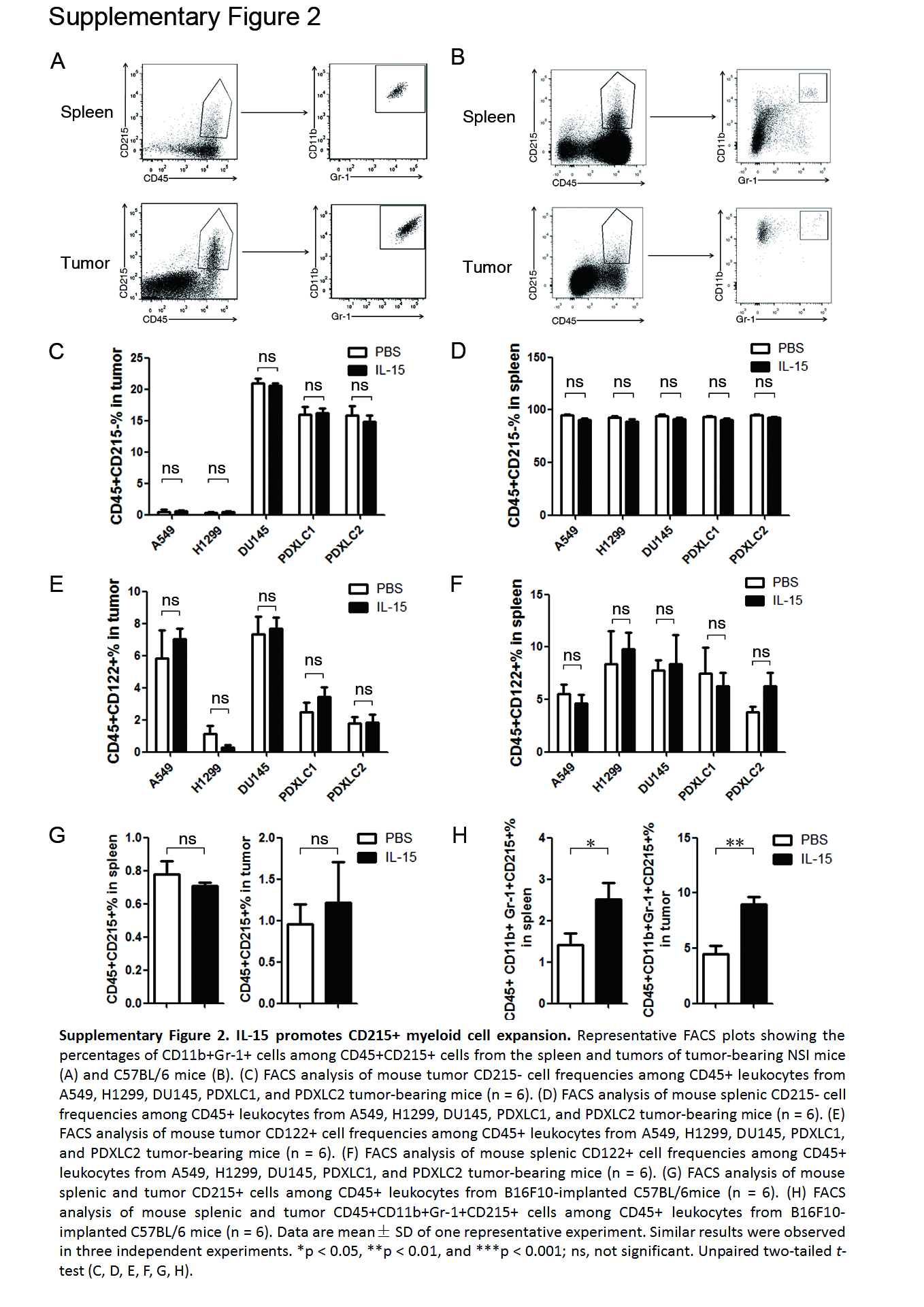

Supplement: Supplementary file 2 [file image_2.tif]

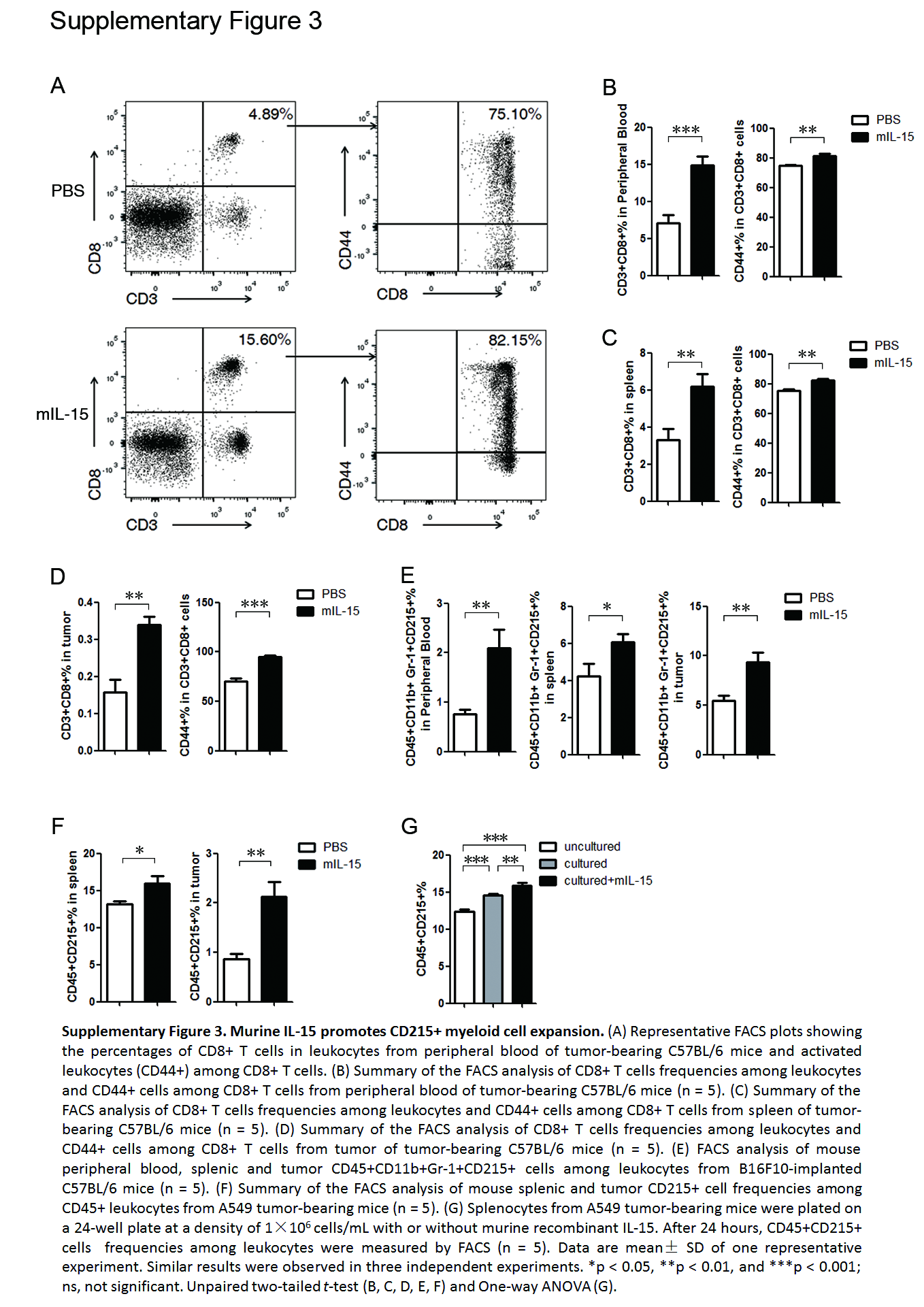

Supplement: Supplementary file 3 [file image_3.tif]

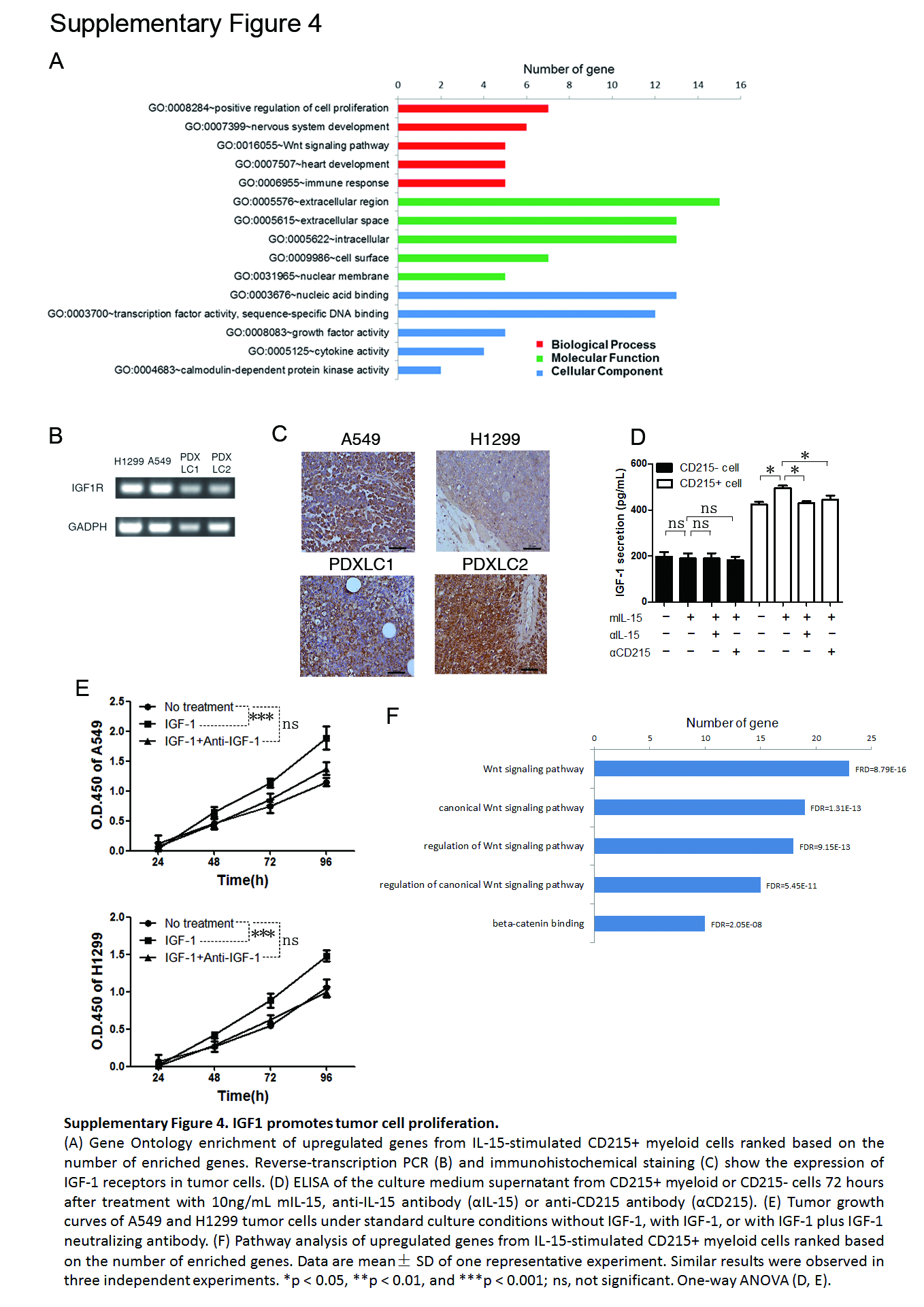

Supplement: Supplementary file 4 [file image_4.tif]
