# Supplementary material for: CD215+ Myeloid Cells Respond to Interleukin 15 Stimulation and Promote Tumor Progression
Source: Front Immunol. 2017 Dec 4;8:1713. doi: 10.3389/fimmu.2017.01713 (PMC5722806; doi:10.3389/fimmu.2017.01713)
Supplement: Supplementary file 5 [file table_1.docx]

**Supplementary table 1.** List of up regulated genes in CD45+CD215+ cells treated with IL15.

| **GeneName** | **CD215-** | | **CD215- IL-15** | | **CD215+** | | **CD215+ IL-15** | |
| --- | --- | --- | --- | --- | --- | --- | --- | --- |
|  | **S1** | **S2** | **S1** | **S2** | **S1** | **S2** | **S1** | **S2** |
| **Il7** | -2.87708 | -2.14717 | -2.22505 | -3.94106 | -0.09596 | -2.27003 | 0.493701 | -0.76434 |
| **Fgf13** | -2.13747 | -0.94465 | -1.61478 | -0.49423 | -1.42605 | -1.04193 | 1.02232 | 1.558534 |
| **Bdnf** | -4.47538 | -4.47538 | -4.47538 | -3.94106 | -3.98548 | -1.9787 | -3.02507 | -1.14311 |
| **Pdgfa** | -1.28771 | 0.72208 | -2.42868 | 0.619308 | 0.685461 | 1.84884 | 1.491771 | 2.656079 |
| **Igf1** | -0.54986 | -2.66241 | -0.41724 | -2.02054 | 0.939025 | 0.910865 | 2.189872 | 1.769076 |
| **Adm** | -3.24155 | -4.47538 | -4.47538 | -4.47538 | 1.166347 | -3.87513 | 2.040813 | -2.46676 |
| **Yap1** | -4.47538 | -3.88779 | -4.47538 | -3.55203 | -4.47538 | -3.12604 | -3.95617 | -1.55853 |
| **Lef1** | -1.95643 | -3.14874 | -2.66585 | -2.42653 | -0.54045 | -1.9787 | 1.0445 | 1.465329 |
| **Sox4** | 1.443093 | 1.246566 | 2.045292 | 1.369604 | 3.077619 | 2.812891 | 4.280948 | 3.498951 |
| **Aff3** | -0.68429 | 0.640768 | 0.119988 | 0.909741 | 1.106709 | -0.34516 | 1.721117 | 0.893374 |
| **Bach2** | -0.68429 | 1.41498 | -0.21511 | 0.780309 | 1.278699 | 0.21208 | 2.390722 | 1.059414 |
| **Hic1** | -1.08811 | -4.47538 | -1.18725 | -3.55203 | 0.701676 | -4.47538 | 1.771359 | -3.88658 |
| **Tchp** | 0.673288 | 0.661529 | 1.108793 | 1.151389 | 1.836482 | 0.65616 | 3.266874 | 2.075137 |
| **Zfp14** | -1.28771 | -0.88322 | -1.0976 | -2.27814 | 0.493917 | -1.9787 | 1.58728 | 0.466464 |
| **Zfp27** | 0.333932 | 2.402768 | 0.043224 | 1.717775 | 2.370442 | 2.350965 | 3.012168 | 3.059601 |
| **Zfp39** | 2.347449 | 1.91608 | 2.504912 | 1.744049 | 2.663197 | 1.927019 | 3.769453 | 2.92405 |
| **Zfp354c** | -4.47538 | -1.38099 | -3.77377 | -4.47538 | -1.29158 | -1.11254 | 0.255623 | -0.46461 |
| **Zfp850** | 1.942368 | 2.124846 | 1.458344 | 2.875714 | 2.186805 | 3.526934 | 2.937124 | 4.192498 |
| **Zscan22** | 2.249438 | 2.717291 | 2.475999 | 3.040246 | 1.721905 | 3.494641 | 2.85019 | 3.9955 |
| **Epcam** | -1.65091 | -0.29804 | -1.74488 | 0.205727 | -0.4076 | -0.67841 | 1.721117 | 0.764615 |
| **Spon1** | -1.18446 | -4.47538 | -1.88789 | -3.94106 | 3.419126 | -1.62903 | 4.517657 | -0.55777 |
| **Serpinb6b** | 1.652809 | -2.46949 | 0.862971 | -2.59195 | 6.623029 | -0.73299 | 7.164136 | -0.1069 |
| **Zmynd15** | -0.11063 | -0.00845 | -0.26305 | 0.780309 | 6.035466 | -0.47868 | 6.613463 | 0.857743 |
| **Serpinb9b** | 0.588365 | -3.88779 | 0.510554 | -4.47538 | 6.352872 | -4.47538 | 7.379535 | -1.46533 |
| **Ttc39a** | -0.83254 | -3.88779 | -0.31264 | -2.99356 | 0.839951 | -0.67841 | 2.50401 | 0.442611 |
| **Il15ra** | 3.590711 | 2.365291 | 3.642689 | 2.937736 | 6.63416 | 4.099322 | 7.57386 | 4.830685 |
| **Itpka** | -2.87708 | -4.47538 | -2.42868 | -3.94106 | -0.59295 | -2.11702 | 0.346807 | -1.46533 |
| **Meis3** | -2.13747 | -2.46949 | -4.47538 | -2.1436 | 2.425177 | -2.4412 | 3.210326 | -1.29524 |
| **Nhlrc1** | -0.91281 | -1.46851 | -1.49545 | -1.70407 | 0.669061 | 0.634767 | 1.32108 | 1.430727 |
| **Gm4980** | -1.65091 | -1.66129 | -4.47538 | -4.47538 | -0.33965 | 0.65616 | 0.552348 | 1.442801 |
| **BC147527** | 0.804582 | 1.260243 | 1.089834 | -0.10221 | 5.879392 | -0.34516 | 6.467451 | 0.36859 |
| **Gm13546** | 2.154163 | 0.661529 | 1.544418 | 0.728613 | 6.035866 | -0.0058 | 6.628306 | 0.910865 |
| **Bcl2l14** | 2.789201 | -0.07559 | 2.961224 | -1.36764 | 7.064856 | -1.18677 | 8.185783 | -0.29461 |
| **Ms4a6b** | 3.473255 | 5.181716 | 4.450606 | 5.229046 | 7.925919 | 3.606015 | 8.4535 | 4.183531 |
| **Ch25h** | -1.18446 | -2.46949 | -2.66585 | -2.42653 | 0.087995 | -1.04193 | 2.296651 | -0.25506 |
| **Igsf23** | -2.34453 | -2.14717 | -1.61478 | -2.1436 | -0.0682 | -1.73644 | 1.036085 | -1.07267 |
| **Nbas** | 1.41048 | 1.024149 | 1.285823 | 1.645276 | 2.899437 | 2.139397 | 3.791938 | 2.680679 |
| **Ppap2a** | -1.79559 | -1.00881 | -2.04664 | -0.53577 | 1.508064 | -1.73644 | 2.609046 | 0.059166 |
| **Dnajb13** | -3.7304 | -2.29935 | -3.30377 | -1.80204 | -0.4076 | -3.12604 | 0.411601 | -2.29652 |
| **Prph** | -4.47538 | -2.88518 | -4.47538 | -3.55203 | -0.44281 | -4.47538 | 0.662942 | -3.46963 |
| **Il10** | -2.34453 | 0.799053 | -4.47538 | 0.501038 | -0.24338 | 1.080546 | 0.956174 | 1.945357 |
| **Aen** | 2.66337 | 2.889974 | 3.032458 | 3.200625 | 4.059816 | 3.831961 | 4.597574 | 4.348253 |
| **Spats1** | -4.47538 | -3.88779 | -3.77377 | -3.94106 | -1.73967 | -4.47538 | -0.5819 | -3.88658 |
| **Efhc1** | -4.47538 | -3.88779 | -4.47538 | -4.47538 | -2.39309 | -3.87513 | 0.432568 | -2.46676 |
| **Dbn1** | -1.18446 | -1.66129 | -0.85788 | -2.02054 | 1.177984 | -1.9787 | 1.999256 | -0.65736 |
| **Gpc4** | -1.95643 | -3.88779 | -1.74488 | -4.47538 | -4.47538 | 0.591004 | -3.57518 | 1.179729 |
| **Rnf122** | -2.34453 | -0.61009 | -1.38524 | -0.37633 | -1.22877 | 0.182897 | 0.533063 | 1.090447 |
| **Nphp3** | 0.558909 | -0.88322 | 0.862971 | -0.53577 | 1.018845 | 0.545873 | 1.882202 | 1.105716 |
| **Nrarp** | -1.95643 | -1.07596 | -2.66585 | -1.09502 | -0.76053 | -0.47868 | 0.608705 | 0.208078 |
| **Tnfrsf11a** | 0.298725 | 5.168178 | 1.089834 | 5.362228 | 4.943211 | 2.290688 | 5.576353 | 3.507599 |
| **Scn4b** | -1.65091 | -4.47538 | -4.47538 | -4.47538 | 0.112459 | -4.47538 | 0.697999 | -1.29524 |
| **Gpnmb** | 5.193803 | 0.924601 | 5.738781 | 0.561385 | 6.995646 | 0.872661 | 7.905537 | 1.978913 |
| **Adck1** | 0.779262 | 0.232609 | 0.64651 | -0.0115 | 2.365362 | 0.349746 | 3.019115 | 1.011578 |
| **Slc39a2** | -0.75651 | -4.47538 | 0.043224 | -4.47538 | 0.854528 | -4.47538 | 1.795838 | -3.14655 |
| **Zfp472** | 2.916591 | 3.952407 | 2.945491 | 4.079928 | 4.139351 | 4.336438 | 4.95451 | 4.917627 |
| **Marco** | -1.95643 | -1.66129 | -2.66585 | -1.80204 | -2.39309 | 0.349746 | -0.54045 | 1.40627 |
| **Hpgds** | 0.148619 | 1.190526 | 0.082116 | 1.138242 | 1.631694 | 1.227338 | 2.706951 | 1.970597 |
| **Fam178b** | 0.067272 | -2.14717 | -0.41724 | -2.27814 | 2.415378 | -3.12604 | 3.266874 | -1.37778 |
| **Gm6904** | -1.18446 | -3.47145 | -0.78608 | -4.47538 | 1.177984 | -4.47538 | 2.22635 | -3.88658 |
| **Ufsp1** | -0.15876 | -1.14638 | 0.36044 | -1.1585 | -0.27476 | 0.296245 | 0.608705 | 1.250129 |
| **Gm16938** | -0.54986 | -1.38099 | -0.41724 | -0.86582 | 0.493917 | -1.11254 | 1.669063 | -0.07213 |
| **Armc9** | -0.42689 | -0.33869 | 0.481755 | -0.86582 | 0.701676 | -0.22295 | 1.97802 | 0.513018 |
| **Htra1** | -1.79559 | 0.836053 | -1.0976 | 0.3722 | 1.535463 | -1.26504 | 2.244249 | -0.4202 |
| **Apof** | -2.87708 | -4.47538 | -3.30377 | -3.94106 | -1.65464 | -4.47538 | -0.71391 | -3.46963 |
| **Ffar4** | -4.47538 | -1.76829 | -3.77377 | -4.47538 | -2.39309 | -2.63546 | -1.65483 | -0.82096 |
| **Pvrl2** | -0.01894 | 0.889831 | 0.566483 | 0.017507 | 2.923833 | -0.84875 | 3.468136 | 0.150344 |
| **Zfp383** | -0.61551 | 1.463079 | -0.93345 | 0.674996 | 1.489505 | 0.475412 | 2.719802 | 1.501702 |
| **Nbl1** | -0.75651 | -1.66129 | -2.66585 | -1.61233 | 1.118836 | -2.4412 | 2.702642 | -0.94133 |
| **Spaca1** | -4.47538 | -4.47538 | -4.47538 | -4.47538 | -3.98548 | -3.87513 | -0.66855 | -3.14655 |
| **Calml4** | -2.34453 | -1.22043 | -1.61478 | -2.42653 | -1.05525 | -1.43558 | -0.05863 | -0.70986 |
| **Gm6484** | -2.58637 | -2.29935 | -2.66585 | -1.44468 | -4.47538 | 0.591004 | -1.94811 | 1.623396 |
| **Kifc3** | 0.067272 | 0.554597 | -0.12376 | 0.846476 | 2.054174 | -0.52608 | 2.649905 | 0.489929 |
| **Fam228b** | -3.7304 | -4.47538 | -4.47538 | -4.47538 | -4.47538 | -2.86 | -1.20287 | -2.14426 |
| **Crb2** | -3.7304 | -4.47538 | -4.47538 | -4.47538 | -2.88 | -4.47538 | -1.94811 | -3.46963 |
| **Ttn** | -0.83254 | 0.365085 | -1.01319 | 0.255282 | 0.228919 | 0.153112 | 1.642314 | 0.745245 |
| **Prickle4** | -3.24155 | -4.47538 | -2.66585 | -3.94106 | -2.39309 | -4.47538 | -1.41122 | -3.14655 |
| **Khdrbs3** | 0.498129 | -0.88322 | -0.71768 | -0.71504 | 0.228919 | 0.182897 | 1.439746 | 0.995273 |
| **Tceal1** | -2.34453 | -1.88387 | -1.88789 | -1.2249 | -1.00176 | -2.4412 | -0.11836 | -0.60671 |
| **Myct1** | -2.87708 | -1.38099 | -3.77377 | -0.41457 | -4.47538 | 0.978351 | -3.95617 | 1.654878 |
| **Fitm2** | 0.498129 | 1.008027 | 0.192873 | 0.045946 | 0.795316 | 1.464079 | 1.490619 | 1.995403 |
| **Krt6a** | -4.47538 | -2.46949 | -3.77377 | -3.55203 | -4.47538 | -1.73644 | -3.27406 | -1.21718 |
| **Rn4.5s** | 2.347449 | 0.486414 | 2.660777 | 0.3722 | 3.017488 | 0.545873 | 3.542352 | 1.059414 |
| **Magi2** | -2.13747 | -0.2585 | -3.77377 | -0.23277 | -0.09596 | -1.62903 | 0.552348 | -0.55777 |
| **Dnaja4** | 0.434674 | -0.11036 | 0.227979 | -2.1436 | 0.475174 | 0.499284 | 1.450065 | 1.84884 |
| **Tmem253** | -3.7304 | -2.88518 | -4.47538 | -4.47538 | -2.53787 | -3.4525 | -1.48794 | -2.29652 |
| **Gbgt1** | -1.95643 | -2.00952 | -2.04664 | -1.61233 | -0.71678 | -2.63546 | 1.651285 | -2.00655 |
| **Znrf3** | -2.34453 | -0.14599 | -1.61478 | 0.416437 | -1.73967 | -0.14688 | -0.71391 | 0.489929 |
| **Bcas1** | -0.9978 | -4.47538 | -1.49545 | -4.47538 | -0.44281 | -4.47538 | 1.008422 | -3.14655 |
| **Tbc1d32** | -0.20854 | 1.132221 | -0.71768 | 1.070644 | 2.586683 | -1.11254 | 3.518035 | -0.17908 |
| **Cdkn2b** | -0.91281 | -0.14599 | -0.08017 | -1.1585 | 3.366702 | -3.87513 | 4.462657 | -2.46676 |
| **Slc25a43** | -0.91281 | 0.799053 | -0.71768 | 0.438057 | 0.939025 | -0.52608 | 2.448478 | 0.535744 |
| **Rbm48** | 2.904932 | 3.854374 | 2.902677 | 3.447105 | 3.509402 | 3.767076 | 4.028943 | 4.287619 |
| **Rbks** | 2.642459 | 2.732089 | 2.588329 | 2.918639 | 2.934163 | 2.936063 | 3.706287 | 3.566724 |
| **Ttc37** | 1.747243 | 1.204742 | 1.70254 | 2.055121 | 0.883247 | 2.564015 | 2.980483 | 3.150753 |
| **Prrt3** | -2.34453 | -3.88779 | -2.94984 | -3.94106 | -1.29158 | -4.47538 | 0.053902 | -1.65818 |
| **Fkbp7** | 0.225623 | -0.56116 | -0.41724 | -0.07134 | 0.037786 | 0.027411 | 1.12893 | 0.978782 |
| **P4htm** | -0.68429 | -3.47145 | -1.0976 | -3.55203 | -0.30684 | -3.4525 | 0.608705 | -2.14426 |
| **Ccdc120** | -2.58637 | -1.66129 | -2.66585 | -1.70407 | 0.01201 | -3.87513 | 0.861544 | -1.88084 |
| **Pf4** | 5.298879 | -0.14599 | 5.86959 | 0.394488 | 4.620744 | 0.854268 | 6.230985 | 1.36879 |
| **Xlr4c** | 0.528839 | 1.451204 | -0.31264 | 0.955433 | 0.456184 | 1.807825 | 2.146114 | 2.430823 |
| **Rragb** | -0.20854 | 1.024149 | -1.38524 | 0.541548 | -0.04096 | 1.138927 | 0.907198 | 1.735618 |
| **Tdrd12** | -3.7304 | -4.47538 | -4.47538 | -3.94106 | -4.47538 | -3.4525 | -1.74612 | -2.46676 |
| **H2-Bl** | 1.595935 | 0.486414 | 1.845031 | 0.501038 | 2.743709 | -0.3883 | 3.290036 | 0.1795 |
| **Layn** | 0.108519 | 0.681997 | 0.452369 | 0.710961 | 0.635692 | 0.37577 | 1.204017 | 0.945223 |
| **Slc6a12** | 1.785896 | 1.313687 | 1.070623 | 1.151389 | 2.192554 | 1.10821 | 3.106515 | 2.172611 |
| **Sgtb** | -0.75651 | -1.14638 | -0.03786 | -0.30276 | 0.925281 | -2.63546 | 1.633286 | -1.65818 |
| **Tbc1d13** | 2.697562 | 2.124846 | 2.504912 | 2.572704 | 2.867453 | 2.131782 | 3.376433 | 2.644543 |
| **Gm15056** | -2.58637 | -0.38053 | -3.77377 | -1.61233 | -1.16858 | -1.62903 | -0.27952 | -0.65736 |
| **Capn5** | -1.18446 | -0.76769 | -3.30377 | -0.30276 | 1.342137 | -3.12604 | 2.627349 | -0.87989 |
| **Calcoco2** | -3.24155 | -3.88779 | -2.66585 | -2.99356 | -3.08725 | -3.87513 | -1.56898 | -1.88084 |
| **Tnfsf10** | 2.479083 | 2.102243 | 2.940208 | 2.0621 | 3.456069 | 1.241999 | 4.398834 | 2.406368 |
| **Gm7444** | -2.34453 | -1.56168 | -1.61478 | -3.24594 | -1.83001 | -1.9787 | 0.749038 | -0.87989 |
| **Camk4** | 0.434674 | 0.836053 | 0.328447 | 1.467466 | 0.717711 | 0.634767 | 2.107521 | 1.263806 |
| **Tjp3** | 2.614098 | 1.565773 | 1.974705 | 2.048109 | 3.406816 | 0.854268 | 4.071338 | 1.591212 |
| **Lnx1** | -1.18446 | -0.14599 | -0.26305 | -0.13376 | -0.95018 | -0.30326 | -0.4228 | 0.393688 |
| **Bicc1** | -4.47538 | -2.29935 | -4.47538 | -3.55203 | -4.47538 | -2.27003 | -3.95617 | -1.00551 |
| **Cachd1** | -4.47538 | -4.47538 | -4.47538 | -4.47538 | -4.47538 | -4.47538 | -1.20287 | -3.46963 |
| **Kcnh2** | -4.47538 | -4.47538 | -4.47538 | -4.47538 | -4.47538 | -4.47538 | -3.57518 | -1.76522 |

“S1” is short for “sample 1”, “S2” is short for “sample 2”
